# Moxifloxacin (Avelox) Induced Thrombotic Thrombocytopenic Purpura

**DOI:** 10.1155/2012/459140

**Published:** 2012-04-11

**Authors:** Sikander P. Surana, Zahily Sardinas, Alan S. Multz

**Affiliations:** Department of Medicine, Nassau University Medical Center, East Meadow, NY 11554, USA

## Abstract

We report a case of a 66-year-old African-American female who presented with complaints of progressively worsening weakness, shortness of breath on minimal exertion, lethargy for the last few days, and short episodes of aphasia lasting 20–30 seconds. Prior to presentation, she was treated with two courses of moxifloxacin for sinusitis. Laboratory examination was remarkable for anemia and thrombocytopenia with elevated lactate dehydrogenase and no evidence of renal failure. Peripheral smear showed numerous schistocytes and she was diagnosed with thrombotic thrombocytopenic purpura. Moxifloxacin was identified as the offending agent. The patient was treated with prednisone and plasmapheresis. To the best of our knowledge, this is the first reported case of thrombotic thrombocytopenic purpura associated with the use of moxifloxacin. Although rare, physicians should be aware of this serious complication associated with its use.

## 1. Introduction

Thrombotic thrombocytopenic purpura (TTP) is characterized by pentad of thrombocytopenia, microangiopathic hemolytic anemia, renal function abnormalities, neurologic symptoms, and fever. It was first described by Moschcowitz in 1925 [1]. Numerous drugs have been implicated in its etiology, the most common being quinine, cyclosporine, ticlopidine, clopidogrel, mitomycin C, bleomycin, cisplatin, gemcitabine and tacrolimus. Rarely fluoroquinolones (especially ciprofloxacin) have also been associated with TTP [2–4]. Fluoroquinolones are synthetic antibiotics widely used due to their broad spectrum coverage, high oral bioavailability, tissue concentrations, and safety profile. We report an unusual case of TTP associated with the use of moxifloxacin (Avelox).

## 2. Case Presentation

**Figure 1:**
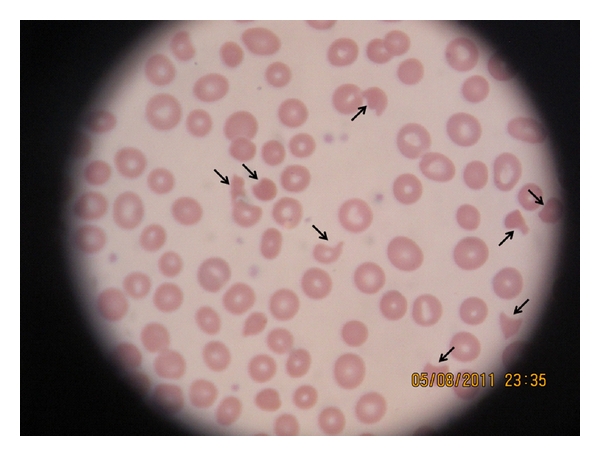
Peripheral smear at admission showing evidence of microangiopathic hemolytic anemia with numerous schistocytes (arrows).

**Figure 2:**
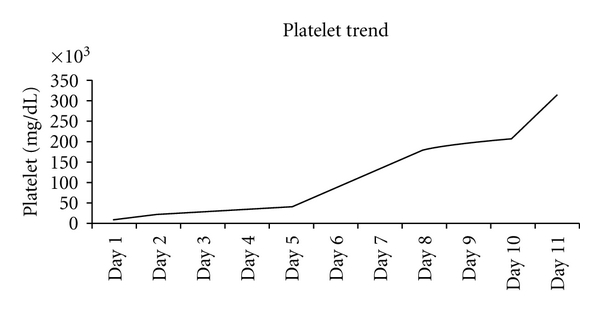
Patient's platelet trend during hospital stay after discontinuation of moxifloxacin and initiation of plasmapheresis.

A 66-year-old African-American female presented to the medical ER with complaints of progressively worsening fatigue, shortness of breath on minimal exertion, and lethargy for the last few days. She also complained of short episodes of aphasia lasting 20–30 seconds. She denied fever, chest pain, sick contacts, blood per rectum, headaches, syncope, or rash. Her past medical history was significant for hypertension, hypothyroidism, osteoarthritis, and gastroesophageal reflux disease. Home medications included synthroid, telmisartan, hydrochlorothiazide, aspirin, omeprazole, moxifloxacin, and fluticasone nasal spray. She denied tobacco, alcohol, or drug use. Prior to presentation, she had been complaining of sinus infection for the last six weeks that was treated with a five-day course of moxifloxacin. Patient's symptoms persisted, and she was started on a second course of moxifloxacin three days prior to presentation. Vital signs were stable. Physical examination was notable for systolic ejection murmur. No petechiae or skin rashes were noted. Neurological exam did not reveal any cranial nerve, motor, or sensory deficit. Laboratory examination was remarkable for anemia (hemoglobin of 5.8 mg/dL) and thrombocytopenia (platelets less than 10,000/mm^3^) with lactate dehydrogenase of 744 U/L. Her serum haptoglobin level was low (<20 mg/dL; normal 30–200 mg/dL) and reticulocyte count was 6.6%. The patient had 22% eosinophils, and peripheral smear revealed numerous schistocytes (Figure 1). Serum creatinine was 1.1 mg/dL (baseline 0.7 mg/dL), and total bilirubin was 1 mg/dL. Chest X-ray and CT scan of the head were unremarkable. A clinical diagnosis of TTP was made. The patient was admitted to medical ICU and received a STAT dose of oral prednisone (1 mg/kg) along with two units of fresh frozen plasma and packed red cells while awaiting plasmapheresis. ADAMTS 13 (a disintegrin-like and metalloprotease with thrombospondin type 1 repeats) level was low. Review of medications suggested that moxifloxacin, which was added recently to the patient's regimen, most likely precipitated TTP. Moxifloxacin was discontinued. A double lumen dialysis catheter was placed and plasmapharesis was initiated and daily plasma exchange was continued. Sepsis was ruled out with negative sets of blood, urine and sputum cultures, no leucocytosis, and absence of fever spikes. The patient showed clinical improvement with plasmapheresis and was subsequently transferred to medical floors. Thrombocytopenia resolved and anemia improved (platelet of 315,000/mm^3^; hemoglobin of 9.7 mg/dL) (Figure 2).

## 3. Discussion

Our patient developed a life-threatening complication related with the use of moxifloxacin. The presence of eosinophilia and recent moxifloxacin use support this. Moxifloxacin is a fluoroquinolone antibiotic approved for treatment of adults (≥18 years old) with acute bacterial sinusitis, acute bacterial exacerbation of chronic bronchitis, community-acquired pneumonia, skin and skin structure infections, and complicated intra-abdominal infections caused by susceptible organisms [5]. Common side effects include nausea, diarrhea, headache, and dizziness. Moxifloxacin is also associated with increased risk of tendinitis and tendon rupture, exacerbation of myasthenia gravis, QT prolongation, hypersensitivity reactions and other serious reactions, including aplastic anemia, agranulocytosis, and pancytopenia [5]. There are a few reports of TTP associated with the use of quinolones (especially ciprofloxacin) [2–4], but to the best of our knowledge, there have been no such reported cases with moxifloxacin.

The mechanism of fluoroquinolone-induced TTP is poorly understood and may be secondary to hypersensitivity reaction. Some authors have postulated that the structural relationship between fluoroquinolones and quinine (quinine differs in the addition of a long side chain at position 4 where quinolones have an oxygen molecule) may play a role [6–8]. These drugs are able to “improve the fit” between weakly autoreactive antibodies complementarity determining regions and target platelet glycoproteins, such as IIb/IIIa [9], thereby improving the structural and chemical interaction. Fluoroquinolone-dependent antibodies against red blood cells [10, 11] and platelets [11–13] have also been described. It is also possible that our patient developed antibodies against moxifloxacin during the initial exposure that led to TTP during subsequent use. However, the exact mechanism of moxifloxacin-induced TTP is uncertain.

Moxifloxacin-induced TTP is extremely rare with our patient being the first reported case in the literature. Hypersensitivity reaction can result in TTP as in our patient. Although rare, physicians should be aware of this serious complication associated with its use.
